# Two weeks of acarbose treatment shows no effect on gut microbiome composition in patients with type 2 diabetes: a randomised, placebo-controlled, double-blind, crossover study

**DOI:** 10.1530/EC-24-0052

**Published:** 2024-06-28

**Authors:** Niels B Dalsgaard, Lærke S Gasbjerg, Laura S Hansen, Dennis S Nielsen, Torben S Rasmussen, Filip K Knop

**Affiliations:** 1 Center for Clinical Metabolic Research, Gentofte Hospital, University of Copenhagen, Hellerup, Denmark; 2 Department of Biomedical Sciences, Faculty of Health and Medical Sciences, University of Copenhagen, Copenhagen, Denmark; 3 Department of Food Science, Faculty of Science, University of Copenhagen, Copenhagen, Denmark; 4 Department of Clinical Medicine, Faculty of Health and Medical Sciences, University of Copenhagen, Copenhagen, Denmark; 5 Steno Diabetes Center Copenhagen, Gentofte, Denmark

**Keywords:** acarbose, alpha-glucosidase inhibitor, gut bacteria, gut microbiome, type 2 diabetes

## Abstract

**Aim:**

The alpha-glucosidase inhibitor acarbose is approved for the treatment of type 2 diabetes (T2D). It acts in the lumen of the gut by reducing intestinal hydrolysis and absorption of ingested carbohydrates. This reduces postprandial blood glucose concentration and increases the content of carbohydrates in the distal parts of the intestine potentially influencing gut microbiome (GM) composition and possibly impacting the gut microbiome (GM) dysbiosis associated with T2D. Here, we investigated the effect of acarbose on GM composition in patients with T2D.

**Methods:**

Faecal samples were collected in a previously conducted randomised, placebo-controlled, double-blind, crossover study in which 15 individuals with metformin-treated T2D (age 57–85 years, HbA1c 40–74 mmol/mol, BMI 23.6–34.6 kg/m^2^) were subjected to two 14-day treatment periods with acarbose and placebo, respectively, separated by a 6-week wash-out period. Faecal samples were collected before and by the end of each treatment period. The GM profiles were evaluated by 16S rRNA gene amplicon sequencing.

**Results:**

The GM profiles after the treatment periods with acarbose or placebo remained unaffected (*P* > 0.7) when compared with the GM profiles before treatment. This applied to the analysis of within-sample diversity (α-diversity) and between-sample bacterial composition diversity (β-diversity). Additionally, no dominant bacterial species differentiated the treatment groups, and only minor increases in the relative abundances of *Klebsiella* spp. and *Escherichia coli* (*P* < 0.05) were observed after acarbose treatment.

**Conclusion:**

In patients with metformin-treated T2D, 14 days of treatment with acarbose showed only minor effects on GM as seen in increased relative abundances of *Klebsiella* spp. and *Escherichia coli*.

## Introduction

In recent years, gut microbial dysbiosis has been recognised as a likely contributor to the development of type 2 diabetes (T2D) (1, 2, 3). It has been proposed that especially members of the *Ruminococcus*, *Fusobacterium*, and *Blautia* genera positively associate with T2D while *Bifidobacterium*, *Bacteroides*, *Faecalibacterium*,* Akkermansia*, and *Roseburia* members negatively associate with and may protect against T2D (4). Acarbose is an alpha-glucosidase inhibitor approved for the treatment of type 2 diabetic postprandial hyperglycaemia (5); typically used as monotherapy in Asia (6) and as an add-on treatment in the rest of the world. The drug competitively inhibits saliva and pancreatic amylase as well as the alpha-glucosidase located in the brush border of the small intestine, thereby limiting carbohydrate digestion and thus absorption of glucose. The resulting propelling of undigested carbohydrates to the distal and L cell-rich parts of the gut augments postprandial L cell secretion of glucagon-like peptide 1 (GLP-1), contributing to the glucose-lowering effect of the drug (7). In addition to its glucose-lowering effect in T2D, the drug reduces the risk of developing T2D in high-risk prediabetic patients (8, 9), and has been shown to induce weight loss in patients with T2D and with metabolic syndrome (10, 11). The mechanisms behind these favourable effects of acarbose are uncertain.

Intake of complex carbohydrates seems to hinder the development of metabolic disease with positive effects on body weight, food intake, glucose homeostasis, and insulin sensitivity (12); perhaps due to a modulating effect on gut microbiome (GM) composition (13). Thus, one could speculate that acarbose-induced propelling of undigested and starch-like carbohydrates distally may drive GM composition in a metabolically favourable direction driving the aforementioned health benefits of acarbose. In line with this notion, several murine studies show the beneficial impact of acarbose on host GM and health (8, 13, 14, 15).

Here, using faecal samples collected in a previously published randomised double-blind, placebo-controlled clinical trial (7, 16), we investigated the effects of a 2-week treatment course with the alpha-glucosidase inhibitor, acarbose, on GM profiles (evaluated by 16S rRNA gene-based high-throughput sequencing) in Caucasian individuals with metformin-treated T2D.

## Materials and methods

### Ethics and study approvals

This study is based on analyses of faecal specimens sampled during a previously published study investigating the effect of acarbose-induced GLP-1 secretion on postprandial glucose metabolism (primary endpoint) in patients with T2D (7). Patients were included after a screening visit where oral and written informed consent was obtained. The study was performed at the Center for Clinical Metabolic Research, Gentofte Hospital, University of Copenhagen, Hellerup, Denmark, after approval by the Ethics Committee of the Capital Region of Denmark (record no. H-17007893). The study was registered at clinicaltrials.gov (ID: NCT03241303) and the Danish Data Protection Agency (local no. HGH-2018-041, I-Suite no. 6.802).

### Research design and experimental procedures

Details of the study design, including materials and methods, have previously been reported (7). In short, we included 15 patients with T2D on metformin monotherapy, age > 18 years, and BMI between 23 kg/m^2^ and 35 kg/m^2^. Study participants were studied in a randomised, double-blind, placebo-controlled, crossover study. For each study participant, the study consisted of two 14-day treatment periods with double-blind administration of either placebo or acarbose (Glucobay, Bayer Pharma AG), with dose escalation to a maximum dose of 100 mg acarbose three times daily (or corresponding placebo tablets) in addition to their usual metformin treatment. At the end of each treatment period, the subjects underwent two liquid mixed meal tests (separated by one full day in between) with double-blind continuous intravenous (iv) infusions of the GLP-1 receptor antagonist exendin(9−39)NH_2_ and isotonic saline, respectively (to describe the effect of acarbose-induced GLP-1 secretion as previously described in detail) (7). The treatment periods were separated by a wash-out period of 6 weeks. All participants collected stool samples at home on the day before the first treatment period and day 13, corresponding to the end of each treatment period. Patients were told to eat a similar diet before the collection of fecal samples and during the treatment periods, but diets were not registered or monitored. Kits containing the necessary collecting equipment (written instruction, cooler bags, sampling tubes, and plastic gloves) were provided to the participants at inclusion, along with careful oral and written instruction on a collection of faeces. The stool sample was stored immediately after sampling in the participant’s freezer and then brought frozen to the hospital in a cooler bag on day 14 and stored at −80°C until analysis.

### DNA extraction and sequencing of the bacterial 16S rRNA gene methods

The Bead-Beat Micro AX Gravity kit (cat. no. 106-100 mod.1, A&A Biotechnology) was used to extract bacterial DNA from the faecal material by following the instructions of the manufacturer. The final purified DNA was stored at −80°C and the DNA concentration was determined using the Qubit HS Assay Kit on the Qubit 4 Fluorometric Quantification device (Invitrogen). Near full-length 16S rRNA gene amplicon sequencing was performed with the MinION platform (Oxford Nanopore Technologies), as previously described (17). In brief, the 16S rRNA gene was amplified by polymerase chain reaction (PCR) with primers targeting conserved regions flanking the hypervariable regions V1−V8. The initial PCR (PCR1) reaction mixture included PCRBIO HiFi polymerase and PCRBIO buffer (PCR Biosystems Ltd.), primer mix, genomic DNA, and nuclease-free water. Gel electrophoresis was used to verify the size of the PCR products that subsequently were barcoded by an additional PCR (PCR2) reaction using the same reagents but with barcoded primers. The final PCR products were purified using AMPure XP beads (Beckman Coulter) and pooled in equimolar concentrations. The pooled barcoded amplicons were ligated according to the 1D genomic DNA using a ligation protocol (SQK-LSK109) to complete library preparation for sequencing on an R9.4.1 flow cell. Data generated by the MinION were collected using MinKnow software v19.06.8. The Guppy v3.2.2 base-calling toolkit was used to base call raw fast5 to fastq. Porechop v0.2.2 was used for adapter trimming and sample demultiplexing (https://github.com/rrwick/Porechop). Sequences containing quality scores (fastq files) were quality corrected using NanoFilt (*q* ≥ 10; read length > 1 kb). Taxonomy assignment of quality-corrected reads against the Greengenes (13.8) database was conducted using the uclast method implemented in parallel_assign_taxonomy_uclust.py (QIIME v1.9.1) (17).

### Calculations and bioinformatic analysis of bacterial sequences

Power calculations for the primary endpoint have been provided in a previously published paper (7). Initially, the dataset was purged for 16S rRNA gene amplicons that were detected in less than 5% of the samples, but the resulting dataset still maintained 99.5% of the total reads. The bacterial diversity (α-diversity) analysis was based on raw read counts and statistics evaluated with ANOVA and *post hoc* analysis with Tukey’s test. Cumulative sum scaling (CSS) normalisation was applied for the analysis of the bacterial composition (β-diversity) (18). Beta-diversity was represented by Bray–Curtis dissimilarity, and statistics based on PERMANOVA and FDR-corrected. Differential abundance was evaluated with DESeq2 using the Wald test for statistics (19). The main packages used in R version 4.01 were metagenomeSeq package (20), phyloseq (21), vegan (22), DESeq2 (19), ampvis2 (23), and ggplot2.

## Results

Fifteen individuals (2 women/13 men) with a median age of 71 (range 57–85) years, weight 87.6 (66.3–111.0) kg, height 1.78 (1.53–1.85) m, body fat mass 35.4 (24.6–45.1)%, BMI 29.7 (23.6–34.6) kg/m^2^, fasting plasma glucose 7.8 (6.6–11.6) mmol/L, HbA1c 48 (40–74) mmol/mol/6.5 (5.8–11.6)% with metformin-treated T2D were studied (7).

### Fecal microbiome analyses

**Figure 1:**
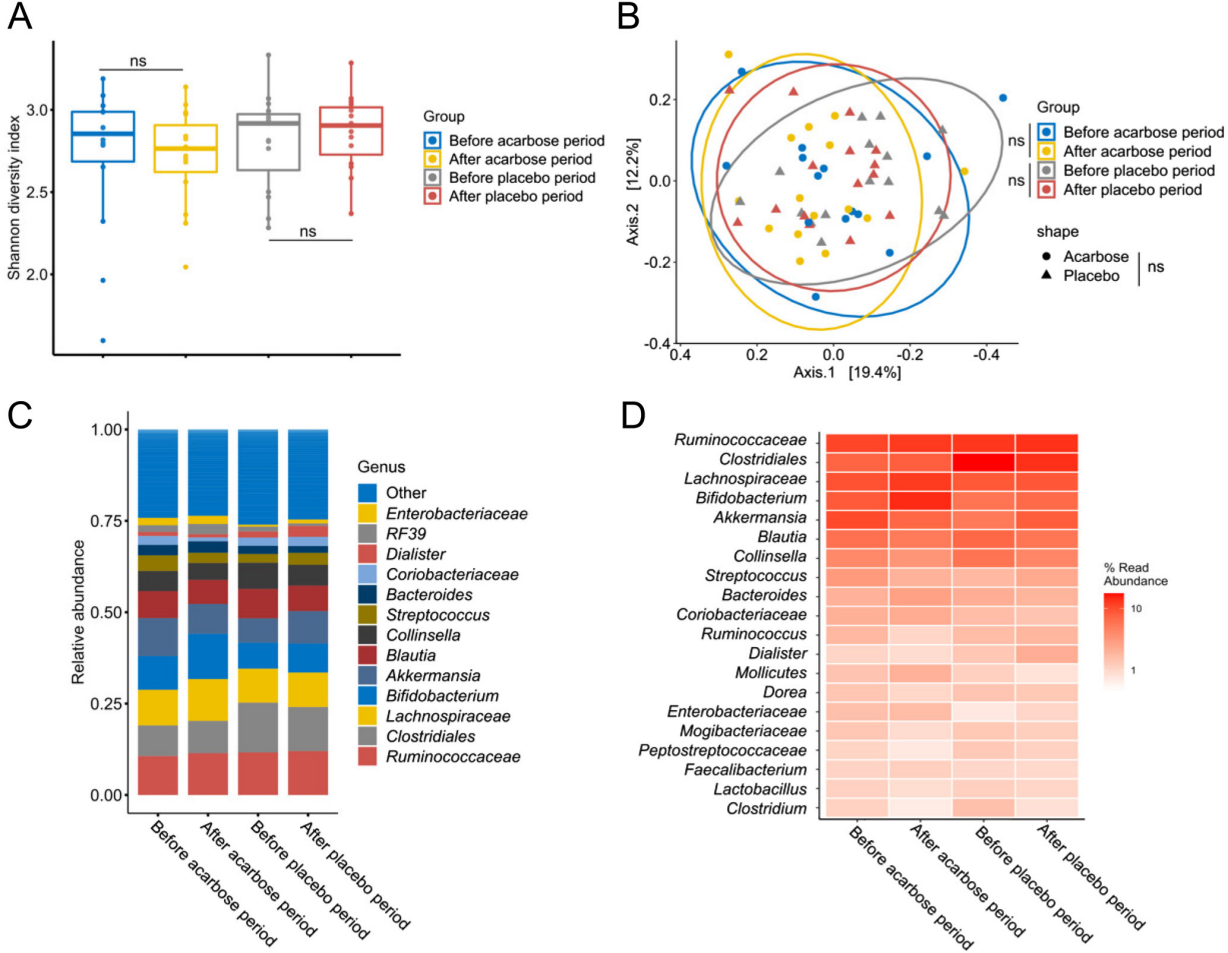
Gut bacteriome analysis of the impact by periods of acarbose or placebo treatment. A) The bacterial diversity based on the Shannon diversity index, B) PCoA plot illustrating the bacterial composition based on Bray–Curtis dissimilarity, C) and D) bar plot and heatmap, respectively, showing the relative abundance distribution of the major bacterial taxa. ‘ns’ = not significant.

**Figure 2:**
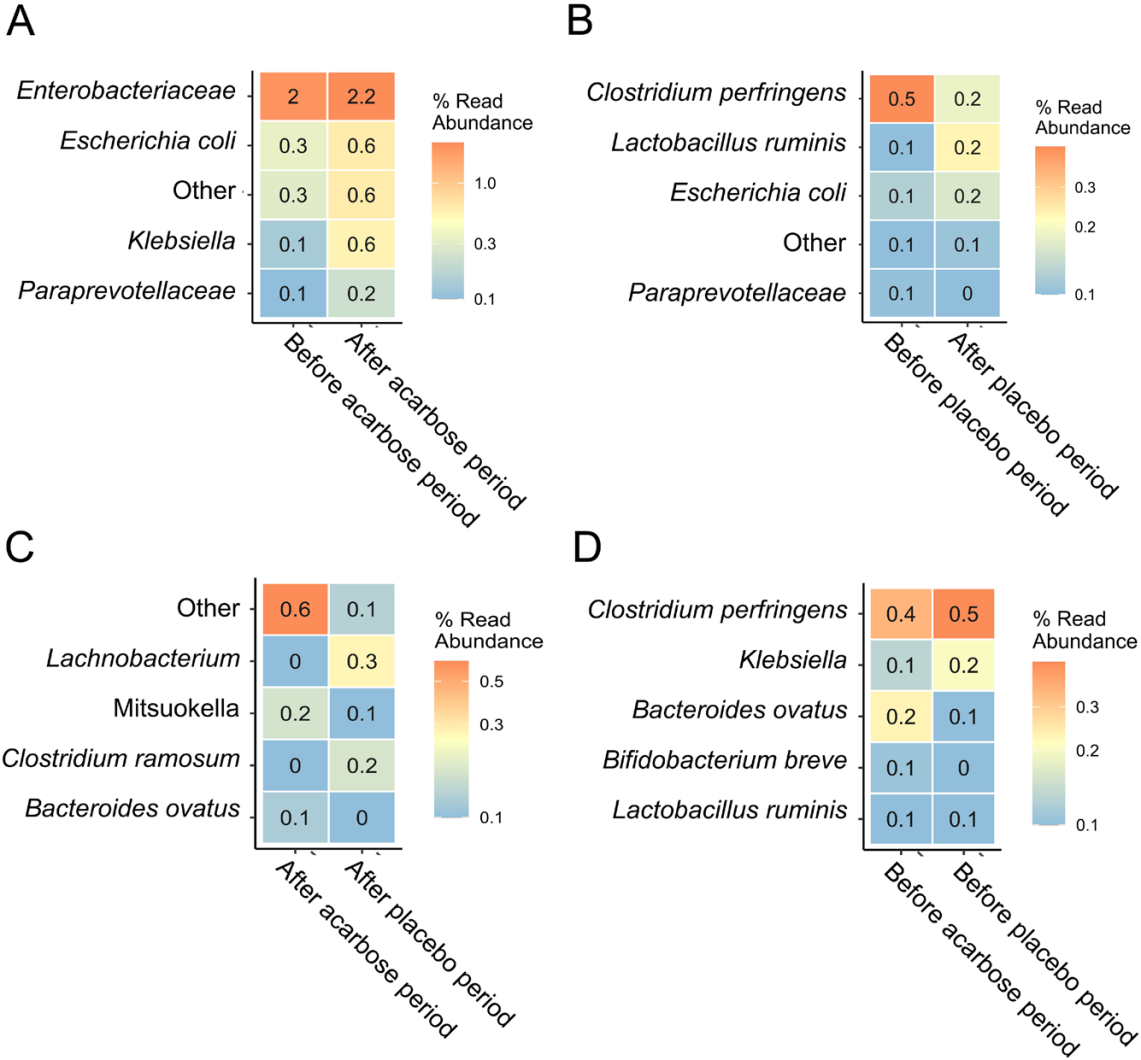
DESeq2-based analysis of the differential relative abundant bacteria with a minimum 2 log_2_ fold difference and with *P* < 0.05, showing top five taxa. Comparing A) before vs after acarbose treatment, B) before vs after placebo treatment, C) after acarbose vs after placebo treatment, and D) before acarbose vs before placebo treatment.

We conducted an analysis to explore potential variations in the GM profiles of individuals resulting from acarbose or placebo treatment, examining both the within-sample bacterial diversity (α-diversity) and compositional differences (β-diversity). The Shannon diversity index (Fig. 1A) did not change with the intervention as neither acarbose nor placebo treatment had a significant impact when comparing before and after treatment (*P* > 0.96). Other α-diversity measures like the number or observed species did not change either (Fig. 2). Furthermore, no significant differences were observed between the acarbose-treated and placebo-treated groups (*P* = 0.14). Similarly, at the compositional level of acarbose treatment, the bacterial composition (Fig. 1B) remained unaffected by both acarbose and placebo treatments when analysing before and after treatment periods (*P* > 0.80). There were no clear differences in the bacterial composition between the acarbose-treated and placebo-treated groups (*P* = 0.70). Dominant bacterial taxa before and after both acarbose and placebo treatments were consistently represented by Ruminoccocaceae, Clostridiales, *Lachnospiraceae*, *Bifidobacterium*, *Akkermansia*, *Blautia*, *Collinsella*, *Streptococcus*, and *Bacteroides* (Figs 1C and D). Analysis of the differential relative abundance of specific bacterial taxa emphasised that the overall GM profiles were unaffected since only minor changes by a few bacterial taxa could be detected (Fig. 2). Particularly, during the acarbose treatment period, the relative abundance of *Klebsiella* spp. increased significantly (*P* < 0.05) from 0.1% to 0.6%, and *Escherichiacoli* increased from 0.3% to 0.6%, compared to their levels before acarbose treatment. Conversely, the placebo treatment period led to a decrease in *Clostridiumperfringens* from 0.5% to 0.2% (Fig. 2) compared with before the placebo treatment.

## Discussion

Several glucose-lowering drugs used to treat diabetes have been speculated to affect T2D-associated GM dysbiosis in a beneficial way, contributing to their mode of action. However, these speculations have mostly been based on observational and associative data and not on data from randomised, placebo-controlled trials (24, 25). Here, analysing faecal samples from a randomised, placebo-controlled clinical study involving patients with metformin-treated T2D, we show that 14 days of treatment with the alpha-glucosidase inhibitor acarbose, known to increase the amount of undigested and complex carbohydrates in the distal parts of the gut, shows only minor effects on the GM composition. We found significant changes in the relative abundance of *Klebsiella* and* Escherichia coli* following acarbose treatment. In addition, we found *C. perfringens* to decrease with placebo treatment. Whether these changes are of clinical relevance remains uncertain as the relative abundance does not contribute to information about the bacterial load of these species (26). Nevertheless, in a clinical setting, *E. coli* has been linked to chronic mucosal inflammation and inflammatory bowel disease (27). Furthermore, studies have shown increased antibiotic resistance among *Klebsiella spp.* including for example carbapenem-resistant (CRKP) and extended-spectrum β-lactam (ESBL)-producing *Klebsiella pneumoniae*, and management of infections is thus becoming increasingly difficult, particularly in neonatal, geriatric, and immunocompromised patients (28). Lastly, *C. perfringens* is a well-known pathogen that causes gastrointestinal infections such as diarrhea and, more severely necrotising enterocolitis (29).

Acarbose has been shown to exert benefits on GM in several murine studies. Interestingly, a recent study in rats suggested that acarbose, via changes in the GM (by decreasing lactobacilli*, Anaeroplasma*,* Adlercreutzia*,* Enterococcaceae bacterium RF39*, and *Corynebacterium* while increasing *Oscillospira*,* Desulfovibrio*, and* Ruminococcus*) regulates Th17/Treg cells in intestinal mucosal immunity, thereby reducing collagen-induced arthritis (8). In another study by Baxter *et al.,* the authors fed mice either a high-starch or high-fibre diet rich in plant polysaccharides together with a small or high dose of acarbose (human-equivalent doses of 15 mg and 240 mg per day, respectively) for 2 weeks. They found only a high dosage of acarbose to affect the GM in both dieting schemes (comparable to our dosing regime) (15). Furthermore, murine faecal amounts of regulatory short-chain fatty acids (SCFAs) (butyrate and propionate, with especially butyrate being associated with better host health) increased. Based on these findings, *Baxteret al.* conclude that only high doses of acarbose lead to significant GM changes and that diet composition impacts the effect of acarbose, as proposed previously (7, 30, 31). In another study in mice, lifelong acarbose treatment-associated changes in faecal bacterial community, and increased abundance of faecal SCFAs (propionate in particular) were suggested to increase life expectancy (14).

Three human studies have previously evaluated the impact of acarbose treatment on GM in Asian populations. In treatment-naive Chinese individuals with prediabetes, 4 weeks of treatment with acarbose (150 mg daily) was shown to increase lactobacilli, *Bifidobacterium* as well as *Faecalibacterium*, *Prevotella*, and *Dialister* (32). A parallel group trial involving Chinese T2D patients randomised to acarbose (150 mg daily for 4 weeks) vs no acarbose added to their regular T2D treatment showed increased abundances of bacterial species with high bile salt hydrolysis activity such as *Bifidobacterium longum* (33) and *Lactobacillus gasseri*, while it reduced the abundance of the secondary bile acid producers* Bacteroides plebeius*, *Bacteroides vulgatus*/*dorei*, and *Clostridium bolteae* (34). Additionally, it was found that acarbose-treated patients showed reductions in circulating lipopolysaccharide and monocyte chemoattractant protein 1 compared to patients not receiving acarbose. Lastly, in a study from 1980, the culture of faeces from Japanese hyperlipidaemic patients sampled before and after 6 weeks acarbose treatment (300 mg daily) showed diminished levels of Enterobacteriaceae, Bacteroidaceae, and lecithinase-positive *Clostridium*, and that acarbose decreases the amount of serum low-density and very-low-density lipoproteins (35). On a different note, the impact of acarbose on GM has been suggested to induce acarbose ‘resistance’. Using biochemical assays, X-ray crystallography, and metagenomic analyses, it has been shown that the GM widespread in Western and non-Western populations had derived specific acarbose kinases, thereby providing microbiome-induced resistance to acarbose (36).

The strengths of the present study include the randomised placebo-controlled double-blind crossover design. The limitations of the present findings include the small number of study participants and that the reported outcomes constitute exploratory, nevertheless predefined, secondary endpoints from a previously published study (7, 16). In addition, it is not known whether longer treatment duration could have a larger impact on the outcome. However, 2 weeks of treatment with acarbose induced changes in murine GM (15). Whether the difference in biological age/development between species (i.e. short lifespan of murine species vs longer lifespan of primates) and time to the introduction of acarbose to their respective gut microbiome remains unknown.

It is noteworthy that microbiota composition in each intestinal segment is different, and the analysis of mucosal rather than faecal microbiota often is more informative to disease pathogenesis or symptom correction. In addition, the enteric bacterial composition of the faecal samples was analysed using 16S rRNA gene sequencing, which limited the taxonomic resolution to the bacterial genus level; thus, the applied analysis cannot account for potentially important changes at the species and strain levels. Nevertheless, the main objective of this study was to evaluate if acarbose causes major changes in the overall bacterial composition, and it was evaluated that the 16S rRNA gene amplicon sequencing was sufficient.

## Conclusion

In patients with metformin-treated T2D, 14 days of treatment with acarbose showed only minor effects on GM, as seen in increased relative abundances of *Klebsiella* spp. and *Escherichia coli*.

## Declaration of interest

NBD, LSG, LSH, and TSR report no conflict of interest that could be perceived as prejudicing the impartiality of the study reported. FKK has served on scientific advisory panels and/or been part of speaker’s bureaus for, served as a consultant to, and/or received research support from 89bio, Amgen, AstraZeneca, Bayer, Boehringer Ingelheim, Carmot Therapeutics, Eli Lilly, Gubra, MedImmune, MSD/Merck, Mundipharma, Norgine, Novo Nordisk, Pharmacosmos, Sanofi, Structure Therapeutics, and Zealand Pharma.

## Funding

The clinical study was conducted at the Center for Clinical Metahttp://dx.doi.org/10.13039/100019827bolic Research, Gentofte Hospitalhttp://dx.doi.org/10.13039/501100002918, University of Copenhagen, Hellerup, Denmark, and supported by Herlev-Gentofte Hospitalhttp://dx.doi.org/10.13039/501100002918’s Research Council; The A.P. Møller Foundation ‘Lægefonden’; the Novo Nordiskhttp://dx.doi.org/10.13039/501100004191 Foundation; and Eva og Hans Carl Adolf Holms Mindelegat. In addition, this work was supported by the Lundbeck Foundation under Grant R324-2019-1880, and the Novo Nordiskhttp://dx.doi.org/10.13039/501100004191 Foundation under Grant NNF-20OC0063874.

## Data sharing

All study data are available for sharing on request to the corresponding author.

## Guarantor statement

All authors agreed to be accountable for all aspects of the work, ensuring that questions related to the accuracy or integrity of any part of the work are appropriately investigated and resolved.

## Author contribution statement

NBD and LSH performed the study. NBD, LSG, and FKK designed the study and wrote the study protocol. FKK conceptualised and academically supervised the study. TSR and DSN generated data. TSR performed the data analyses. NBD, LSG, TSR, DSN, and FKK drafted the manuscript. All authors contributed substantially to the acquisition, analysis, and/or interpretation of data for the work, critically revised the manuscript for important intellectual content, and approved the final version of the manuscript.
